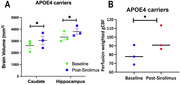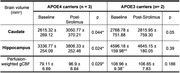# Short‐term Sirolimus Treatment Restores Hippocampus and Caudate Volumes and Global Cerebral Blood Flow in Asymptomatic APOE4 Carriers Compared with Non‐carriers

**DOI:** 10.1002/alz.092991

**Published:** 2025-01-09

**Authors:** Abeoseh Flemister, Xin Xing, Jessica Overschmidt, Joanne Cassani, Stefan J. Green, David Q. Beversdorf, Carter Woods, John W. Grinstead, Talissa A. Altes, Ai‐Ling Lin, Maalavika Govindarajan

**Affiliations:** ^1^ University of Missouri, Columbia, MO USA; ^2^ University of Kentucky, Lexington, KY USA; ^3^ Rush University, Chicago, IL USA; ^4^ Siemens Healthineers, Columbia, MO USA; ^5^ University of Missouri ‐ Columbia, Columbia, MO USA

## Abstract

**Background:**

Apolipoprotein ε4 allele (APOE4) is the strongest genetic risk factor for late‐onset Alzheimer’s disease (AD). Compared with non‐carriers, cognitively normal APOE4 individuals have shown brain atrophy and lower cerebral blood flow (CBF) decades before AD pathological and clinical symptoms appear. The goal of the study is to determine if using Sirolimus, an FDA‐approved mTOR inhibitor, could restore the brain volumes in structures related to cognitive functions and global CBF (gCBF) for asymptomatic APOE4 carriers compared with non‐carriers.

**Method:**

The study was performed at the University of Missouri. Low dose Sirolimus (1 mg/day) was given for 4 weeks to three middle‐aged, cognitively normal APOE4 carriers (F:M = 2:1) and two female APOE3 individuals (45‐65 yrs; MOCA > 27). Oral swabs were used to determine APOE status. 3T MRI‐based structural MPRAGE and T1‐weighted images, and pseudo‐continuous arterial spin‐labeled (PCASL, 1.7x1.7x4mm resolution) were acquired at baseline (pre‐treatment) and Post‐Sirolimus (the end of treatment). FreeSurfer 7.4 was used to automatically generate segments for volumetric analysis and PCASL images were analyzed via MANGO software for perfusion weighted gCBF.

**Result:**

APOE4 carriers had significantly increased Caudate and Hippocampal volumes (Figure 1A) and gCBF (Figure 1B) after 4 weeks of Sirolimus treatment. These differences were not found in the non‐carriers (APOE3 participants). The quantitative data is shown in Table 1. It also shows that APOE4 carriers had significantly lower Hippocampal volume and gCBF at baseline compared with that of APOE3 participants (indicated by “**”), and Sirolimus tends to restore the values to closer to those of the non‐carriers. No side effects were observed, and no changes in blood glucose and HbAc1 levels were found in all the participants.

**Conclusion:**

We show that short‐term Sirolimus treatment can effectively restore brain volumes and gCBF for asymptomatic APOE4 carriers. Specifically in the caudate and hippocampus, which play many roles integral in normal cognitive functioning including memory, learning, and emotional regulation, has considerable atrophy in AD. The findings imply that Sirolimus may be useful and effective to mitigate or prevent AD developments for asymptomatic APOE4 carriers.